# Exploring Metabolic Signatures: Unraveling the Association with Obesity in Children and Adolescents

**DOI:** 10.3390/nu17111833

**Published:** 2025-05-28

**Authors:** Diamanto Koutaki, Garyfallia Stefanou, Sofia-Maria Genitsaridi, Eleni Ramouzi, Athanasia Kyrkili, Meropi D. Kontogianni, Eleni Kokkou, Eleni Giannopoulou, Penio Kassari, Evangelia Charmandari

**Affiliations:** 1 Center for the Prevention and Management of Overweight and Obesity in Childhood and Adolescence, Division of Endocrinology, Metabolism and Diabetes, First Department of Pediatrics, National and Kapodistrian University of Athens Medical School, ‘Aghia Sophia’, Children’s Hospital, 11527 Athens, Greece; mk_madw@hotmail.com (D.K.); sgenitsaridi@gmail.com (S.-M.G.); eleni_ramouzi@hotmail.gr (E.R.); g.elenaki@ymail.com (E.K.); gia_eleni@yahoo.gr (E.G.); peniokassari@gmail.com (P.K.); 2 ECONCARE—Health Research & Consulting, 11528 Athens, Greece; g_stefanou@hotmail.com; 3 Department of Nutrition and Dietetics, School of Health Sciences and Education, Harokopio University of Athens, 17671 Athens, Greece; akirkili@hua.gr (A.K.); mkont@hua.gr (M.D.K.); 4 Division of Endocrinology and Metabolism, Center of Clinical, Experimental Surgery and Translational Research, Biomedical Research Foundation of the Academy of Athens, 11527 Athens, Greece

**Keywords:** metabolomics, metabolic signatures, metabolic biomarkers, childhood obesity

## Abstract

**Background:** Childhood obesity is a growing global health concern. Metabolomics, the comprehensive study of metabolites within biological systems, offers a powerful approach to better define the phenotype and understand the complex biochemical alterations associated with obesity. The aim of this systematic review was to summarize current knowledge in the field of metabolomics in childhood obesity and to identify metabolic signatures or biomarkers associated with overweight/obesity (Ov/Ob) and Metabolically Unhealthy Obesity (MUO) in children and adolescents. **Methods:** We performed a systematic search of Medline and Scopus databases according to PRISMA guidelines. We included only longitudinal prospective studies or randomized controlled trials with ≥12 months of follow-up, as well as meta-analyses of the above that assessed the relation between metabolic signatures related to obesity and Body Mass Index (BMI) or other measures of adiposity in children and adolescents aged 2–19 years with overweight or obesity. Initially, 595 records were identified from PubMed and 1565 from Scopus. After removing duplicates and screening for relevance, 157 reports were assessed for eligibility. From the additional search, 75 new records were retrieved, of which none were eligible for our study. Finally, 7 reports were included in the present systematic review (4 reporting on Ov/Ob and 4 on MUO). **Results:** The presented studies suggest that the metabolism of amino acids and lipids is primarily affected by childhood obesity. Metabolites like glycoprotein acetyls, the Apolipoprotein B/Apolipoprotein A-1 ratio, and lactate have emerged as potential biomarkers for insulin resistance and metabolic syndrome, highlighting their potential value in clinical applications. **Conclusions:** There is a need for future longitudinal studies to assess metabolic changes over time, interventional studies to evaluate the efficacy of therapeutic strategies, and large-scale population studies to explore metabolic diversity across different demographics. Our findings reveal specific biomarkers in the amino acid and lipid pathway that may serve as early indicators of childhood obesity and its associated cardiometabolic complications.

## 1. Introduction

Obesity has emerged as a significant global health issue, with its prevalence having nearly tripled from 1975 to 2016 [1]. According to the World Health Organization (WHO), approximately 60% of adults in Europe will be overweight or obesity in 2022 [2]. This alarming trend not only poses immediate health risks but also predisposes affected subjects to long-term health complications, such as hypertension, left ventricular hypertrophy, insulin resistance and diabetes mellitus type 2 (DM2), metabolic dysfunction-associated steatotic liver disease (MASLD), as well as mental health issues and cancer [3]. Furthermore, during the last decade, obesity with or without metabolic aberrations, commonly termed Metabolically Unhealthy Obesity (MUO) or Metabolically Healthy Obesity (MHO), respectively, has been extensively investigated [4]. Metabolically Unhealthy Obesity (MUO) refers to subjects with obesity who exhibit metabolic abnormalities, such as insulin resistance, elevated blood pressure, dyslipidemia (elevated triglycerides and low HDL cholesterol concentrations), and chronic inflammation. Unlike metabolically healthy obesity (MHO), where subjects have excess body fat but normal metabolic profiles, MUO is strongly associated with an increased risk of DM2, cardiovascular disease, and non-alcoholic fatty liver disease (NAFLD).

The intricate interplay of genetic, epigenetic, environmental, and lifestyle factors contributes to the multifaceted nature of this epidemic [3]. In the quest for a better understanding of the underlying mechanisms and potential interventions, metabolomics has emerged as a powerful tool, offering insights into the metabolic alterations associated with childhood obesity [5,6].

Metabolomics is a field of study within the broader discipline of systems biology, which focuses on the comprehensive analysis of small molecules or metabolites (<1500 KDa) in a biological sample [7,8]. Metabolites are the end products of cellular processes, and their concentrations can provide insights into the biochemical pathways and physiological status of an organism at a specific point in time. It is particularly useful in understanding the dynamic responses of biological systems to various alterations, including genetic, epigenetic, or protein-level modifications, exposure to environmental factors (physical exercise, diet, and microbiome) and diseases, and helps bridge the gap between genotype and phenotype.

The primary goal of metabolomics is to profile and quantify the complete set of metabolites present in a biological sample, such as blood, urine, or tissues. This profiling involves the use of advanced analytical techniques, such as mass spectrometry (MS) and nuclear magnetic resonance (NMR) spectroscopy, coupled with various chromatographic separations to identify and quantify the diverse array of metabolites [9]. This way, the systematic study of small molecules within biological systems may help us gain insight into the metabolic changes associated with obesity, such as adipocyte-related inflammation and insulin resistance [10]. The in-depth study of the unique metabolic fingerprints associated with obesity may help us identify potential biomarkers and altered metabolic pathways, and discover novel therapeutic targets. Numerous studies have underscored the utility of metabolomics in elucidating the complex interplay between genetic predisposition, dietetic habits, gut microbiome, and environmental factors in the pathogenesis of obesity in adults [11]. However, to the best of our knowledge, few studies have been conducted in children and adolescents [12]. Metabolic signatures may differ in early life, given that children do not usually receive medical treatment for obesity. Therefore, metabolomic profiling in children and adolescents will not only facilitate the identification of potential biomarkers for the prevention and management of childhood obesity and its associated complications, but it will also unravel novel therapeutic targets.

The aim of this systematic review was to summarize the current knowledge on metabolomics, childhood obesity, and MUO, and to identify metabolic signatures or biomarkers associated with obesity in children and adolescents, thereby offering a comprehensive analysis of studies that employ metabolomic approaches. Through critical examination of the literature, this review aims to gain a better understanding of the metabolic intricacies associated with childhood obesity and inform the direction of future research and therapeutic strategies.

## 2. Materials and Methods

### 2.1. Study Design

This systematic literature review (SLR) was conducted following the Preferred Reporting Items for Systematic Reviews and Meta-Analyses (PRISMA) protocol [13]. The objectives were formulated using the PICO/PECO (Population, Interventions/Exposure, Comparators, Outcomes) framework (Table 1). The review was registered in the International Prospective Register of Ongoing Systematic Reviews (PROSPERO 2023 CRD42023494461; https://www.crd.york.ac.uk/prospero/display_record.php?ID=CRD42023494461, accessed on 29 December 2023).

### 2.2. Eligibility Criteria

The review included longitudinal prospective studies and randomized controlled trials (RCTs), with a minimum of 12 months of follow-up, and meta-analyses of the above in order to ensure a better quality of methodological design, which would also allow etiological assumptions. The studies examined the metabolic signatures related to obesity, Body Mass Index (BMI), or/and other measures of adiposity and MUO in children and adolescents aged 2–19 years with overweight or obesity. The language was restricted to English and the geographic location included only Western countries (Europe, USA, Canada, and Oceania) that share similar socioeconomic, physical, and dietary environments. The inclusion and exclusion criteria are shown in Table 2.

### 2.3. Literature Search

A comprehensive literature search was conducted using PubMed and Scopus databases for studies published from 1 May 2023 to 16 September 2023. An additional data search was performed on 3 July 2024 to update the results, retrieving studies published after 16 September 2023. The search strategy included a complex string of keywords related to metabolic biomarkers, obesity, adiposity, and associated metabolic and endocrine disorders in children and adolescents. The detailed search strings used for MEDLINE (PubMed) and Scopus are presented in the Supplementary Materials, File S1.

### 2.4. Study Selection

Two independent researchers (GS and DK) screened the records identified from the databases. In instances of disagreement, a third researcher (EC) conducted a final review.

### 2.5. Data Extraction, Outcomes, and Data Synthesis

Relevant data from eligible studies were extracted, including publication details, study design, sample size, participant characteristics, metabolic signatures, biomarkers assessed, and outcomes related to obesity and metabolic disorders. The primary outcomes assessed were obesity and MUO in pediatric populations.

### 2.6. Validity Assessment

All included studies were assessed for risk of bias using the Risk Of Bias In Non-randomized Studies—of Exposures (ROBINS-E) tool [14]. The risk of bias for each study was evaluated across seven domains: confounding (D1), measurement of exposure (D2), selection of participants (D3), post-exposure interventions (D4), missing data (D5), measurement of outcomes (D6), and selection of reported results (D7). Three of the included studies were post hoc analyses of participants who underwent a lifestyle intervention (“Obeldicks”) within a non-randomized controlled trial [15,16] or a double-blind, randomized intervention trial [17]. Since the exposure of interest (metabolites) was not actively assigned, ROBINS-E was deemed the most appropriate tool for assessing the risk of bias in these studies.

### 2.7. Data Management and Synthesis

Data were managed using Mendeley and Excel. Data extraction forms were piloted and refined to ensure consistency and accuracy. Discrepancies between reviewers were resolved through discussion and consensus. The extracted data were synthesized to provide a comprehensive analysis of the metabolic signatures associated with childhood obesity and MUO. The synthesis involved qualitative analyses to summarize the findings and identify potential biomarkers and therapeutic targets. The characteristics of the included studies, e.g., study design, country, sample size, age, follow-up period, methodology, key metabolites identified, and reported outcome are presented in Table 3.

### 2.8. Ethical Considerations

Since this study is a systematic review, ethical approval was not required. However, ethical standards were maintained throughout the review process, ensuring the integrity and accuracy of the findings.

## 3. Results

### 3.1. Characteristics of Included Studies

**Figure 1:**
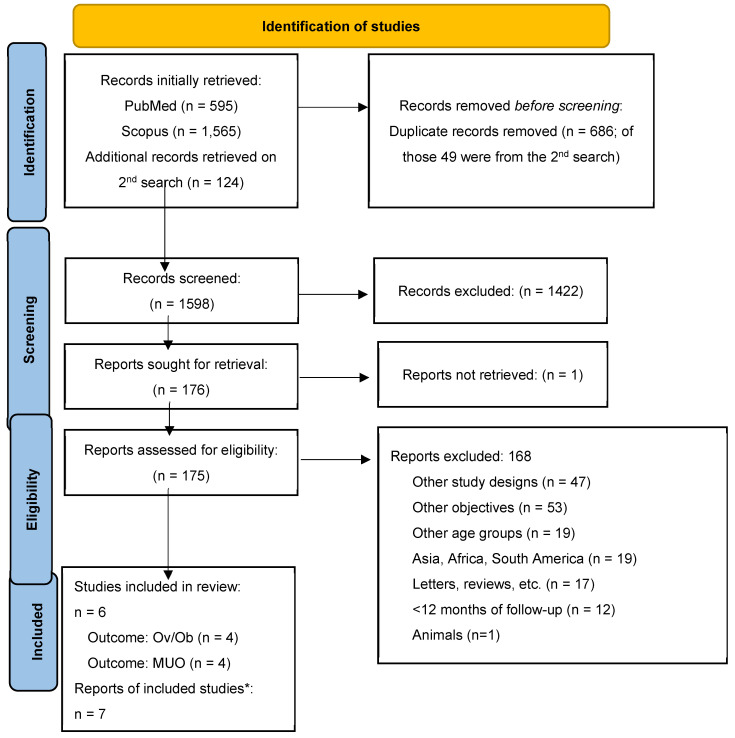
PRISMA flow diagram. Notes: Ov/Ob = overweight/obesity; MUO = Metabolically Unhealthy Obesity. * Two reports were referred to a single study.

Initially, 595 records were identified from PubMed and 1565 from Scopus. After removing duplicates and screening for relevance, 175 reports were assessed for eligibility. Ultimately, 7 (4 longitudinal and 3 post hoc analyses of interventional studies) reports were included in the review. From the additional search, 124 new records were retrieved, from which none were eligible for our study. The flow diagram is presented in Figure 1.

Seven reports, which were derived from six studies, met the inclusion criteria and were included in this systematic review. These studies were conducted in various countries, including the USA [18], Germany [15,16,17], Belgium [17], Italy [17], Poland [17], Spain [17], Australia [19], UK [21], Switzerland [21] and Finland [20]. The included studies were primarily longitudinal cohort studies [18,19,20,21] and post hoc analyses of intervention studies [15,16,17], with follow-up periods ranging from 1 year [15,16,18] to 11 years [21]. The intervention part included lifestyle recommendations regarding physical activity, nutrition, and behavioral therapy for the children and their families. The sample size of these studies varied significantly, from 40 to 396 participants, and the age at baseline ranged from 2 to 19 years. Serum and urine samples were used for the assessment of metabolomic biomarkers.

The included studies examined the association between metabolic profiles and obesity-related outcomes using various approaches (Table 4). Singh et al. (2023) explored the metabolic features associated with increased BMI at one-year follow-up [18]. Mansell et al. (2022) explored the association between changes in BMI and metabolomic profiles over a 5.5-year follow-up period [19]. Reinehr et al. (2014) assessed the metabolite changes in children with obesity who underwent a lifestyle intervention and compared those with substantial weight loss to those without substantial weight loss [15]. Hellmuth et al. (2019) used metabolite concentrations at 5.5 years to predict BMI z-scores at age 8 in the CHOP study [17]. Ojanen et al. (2021) developed a standardized risk score for metabolic syndrome (MetS), having incorporated metabolic and cardiovascular parameters that confer cardiometabolic risk [20]. Hellmuth et al. (2016) investigated the association between metabolite changes and HOMA-IR over a one-year lifestyle intervention (physical activity, nutrition education, and behavior therapy) [16]. Hosking et al. (2019) examined the relation between individual metabolites and insulin resistance (HOMA-IR) in healthy children, taking into account the effects of age, BMI, growth, puberty, adiposity, and physical activity [21]. This study included a pilot phase to identify metabolically distinct profiles related to insulin resistance and a follow-up phase extending the analysis to the age of 16 years to validate the findings.

### 3.2. Obesity Outcomes and Their Association with Metabolites

Our findings suggest that weight gain and weight loss influence distinct metabolic pathways, particularly in amino acids, lipids, and glycolysis-related metabolites.

#### 3.2.1. Amino Acids and Obesity Outcomes

Changes in amino acid metabolism were consistently observed across studies. Singh et al. (2023) reported increases in Citrulline, 4-hydroxyproline, and Inosine with BMI gain [18]. Mansell et al. (2022) found that BMI reduction was associated with decreases in alanine, phenylalanine, and tyrosine [19]. In contrast, Reinehr et al. (2014) identified higher levels of glutamine and methionine in children who experienced significant weight loss [15]. These findings suggest that weight loss is associated with increased levels of certain amino acids (e.g., glutamine, methionine), while weight gain may correspond to increases in other amino acids (e.g., Citrulline, hydroxyproline).

#### 3.2.2. Lipids and Fatty Acids and Obesity Outcomes

Metabolomic shifts in lipid profiles were evident. Mansell et al. (2022) observed that BMI reduction led to decreases in VLDL cholesterol and monounsaturated fatty acids (MUFAs), alongside increases in polyunsaturated fatty acids (PUFAs), Omega-6, and HDL cholesterol [19]. Similarly, Reinehr et al. (2014) found significant changes in phospholipids (PCaeC34:1, PCaeC34:2, etc.) among children who lost weight [15]. In contrast, Hellmuth et al. (2019) reported a positive association between sphingomyelins (SM 32:2, SM 34:2) and free carnitine with BMI at the age of 8 years, although this association was not as strong after adjusting for BMI at the age of 5.5 years [17]. These results indicate that weight gain is associated with sphingomyelins and free carnitine, whereas weight loss is associated with phospholipid alterations and shifts in fatty acid composition (e.g., increased PUFAs and Omega-6 levels).

#### 3.2.3. Glycolysis-Related and Energy Metabolites and Obesity Outcomes

Metabolites involved in energy metabolism were also affected by weight changes. Mansell et al. (2022) demonstrated that BMI reduction was associated with lower pyruvate levels, suggesting altered glycolysis [19]. In addition, Singh et al. (2023) found that 3’-Sialyllactose increased with increases in BMI, potentially indicating metabolic shifts related to energy balance [18]. These findings suggest that an increase or decrease in BMI influences glycolysis and energy-related pathways, with weight reduction linked to decreased glycolysis activity (e.g., lower pyruvate) and weight gain associated with increased sialyllactose levels.

### 3.3. Metabolically Unhealthy Obesity Outcomes and Their Association with Metabolites

#### 3.3.1. Apolipoproteins, Lipids, and Fatty Acids in Relation to Risk for Metabolic Syndrome

Lipid-related biomarkers have been consistently associated with metabolic dysfunction. Ojanen et al. (2021) found that higher ApoB/ApoA ratios and glycoprotein acetyls (GlycAs) were strong predictors of metabolic syndrome, whereas higher large high-density lipoprotein phospholipids (L-HDL-PLs) were protective, showing a negative association with MetS [20]. Similarly, findings from the CHOP study (Hellmuth et al., 2019) demonstrated that higher levels of non-esterified fatty acids (NEFAs 26:1, 26:2, 26:3) were positively associated with HOMA-IR, even after adjusting for BMI, indicating their potential role in insulin resistance [17]. These findings suggest that increased ApoB/ApoA, GlycAs, and NEFAs contribute towards a greater metabolic risk, while HDL phospholipids may play a protective role.

#### 3.3.2. Amino Acids and Insulin Resistance

Amino acid metabolism has also been associated with insulin sensitivity and metabolic health. Hellmuth et al. (2019, CHOP study) found that higher glutamine levels were associated with lower HOMA-IR, suggesting a protective effect against insulin resistance [17]. In contrast, findings from the EarlyBird study (Hosking et al., 2019) indicated that multiple amino acids, including leucine, valine, alanine, and glutamine, were associated with insulin resistance [21]. In addition, Hellmuth et al. (2016) reported that weight loss resulted in increases in proline, tyrosine, and valine, along with acylcarnitine shifts, but decreases in Carn C6:1-DC and Carn C6-oxo, suggesting improved insulin sensitivity [16]. Taken together, these findings indicate that higher glutamine levels may be beneficial, whereas BCAAs (leucine, valine) are associated with metabolic dysfunction.

#### 3.3.3. Energy Metabolism and Insulin Sensitivity

Metabolic markers related to energy metabolism, particularly glycolysis byproducts and mitochondrial metabolites, further illustrate the metabolic shifts associated with MUO. The EarlyBird study (Hosking et al., 2019) identified a strong positive association between lactate and insulin resistance, reinforcing its role as a key metabolic indicator of metabolic health [21]. Furthermore, Hellmuth et al. (2016) demonstrated that acylcarnitine profiles shifted in response to weight loss, with increases in Carn C0, Carn C3, and Carn C5, and decreases in Carn C6:1-DC and Carn C6-oxo levels, reflecting changes in mitochondrial fatty acid oxidation associated with improved insulin sensitivity [16]. Collectively, these findings may suggest that higher lactate levels are strongly associated with insulin resistance, while shifts in acylcarnitine metabolism may reflect improved mitochondrial function following weight loss.

The above results, as well as the statistical analysis performed, are presented in Table 4.

### 3.4. Risk of Bias Assessment

The studies by Singh et al., 2023 [13], and Reinehr et al., 2015 [15], have a high overall risk of bias mainly due to concerns in domains D3 and D1, respectively. The study by Hellmuth et al., 2019 [16], has a high overall risk of bias mainly due to concerns in domain D1. The other studies were of some concern regarding the overall risk of bias, mostly due to issues in domains D3 or D5. The overall risk of bias for each study is summarized in Table 5.

## 4. Discussion

Obesity is a significant and escalating health problem globally, impacting both adults and children. Despite its prevalence, the precise mechanisms driving the development of obesity in children remain unclear. Therefore, in our systematic review, we aimed to identify candidate metabolites or profiles of obesity in children and adolescents who are relatively free of its metabolic complications. To the best of our knowledge, this is one of the first systematic reviews summarizing all available longitudinal studies focusing on metabolomics in childhood obesity.

Our thorough investigation of currently available longitudinal studies demonstrated that childhood obesity is associated with unique alterations in the metabolome, especially in lipid and amino acid metabolism. From a longitudinal perspective, our results strengthen the conclusions from other reviews that included cross-sectional data [11,12,22]. It is well known that obesity affects lipid levels through various lipid metabolism processes, including fatty acid oxidation, lipolysis, and lipogenesis [23]. Furthermore, numerous lipids act as signaling molecules in inflammation pathways or insulin resistance, contributing to obesity-related complications, such as DM2 and cardiovascular disease. Acylcarnitines are the byproducts of noncomplete fatty acid oxidation [24]. The majority of the included studies stated an association of lipids with changes in BMI, insulin resistance, and the risk of metabolic syndrome [15,17,19,20]. More specifically, these included certain lipoproteins (XL-VLDL-L, L-VLDL-L, and S-VLDL-L); Apolipoproteins (ApoB/ApoA1); cholesterols (VLDL-C); fatty acids (MUFAs, MUFAs%); glycerides and phospholipids (total triglycerides, VLDL-TGs, and TG/PG); and increases in certain cholesterols (HDL-C, HDL2-C), fatty acids (unsaturation, LA%, Omega-6%, and PUFAs), ketone bodies (Acetoacetate, 3-hydroxybutyrate), the lyso-phosphatidylcholines LPCaC18:1, LPCaC18:2, and LPCa20:4, the acyl–alkyl phosphatidylcholines PCaeC36:2, NEFAs 26:1, 26:2, and 26:6 l; and L-HDL-PLs. The study by Mansell et al. [19] provides a detailed overview of how weight loss can affect a wide array of lipoproteins, fatty acids, and amino acids. For instance, increases in unsaturated fatty acids (LA%, PUFAs%) and HDL cholesterol are indicative of an improvement in lipid metabolism, which is generally associated with better insulin sensitivity. Conversely, certain lipoproteins (like VLDL-C) and saturated fatty acids (MUFAs%) may reflect adverse metabolic changes. These results are consistent with findings from Ojanen et al. [20], who identified the Apolipoprotein B/Apolipoprotein A-1 ratio and glycoprotein acetyls as predictors of metabolic syndrome, reinforcing the concept that changes in lipoproteins and fatty acid composition may serve as useful biomarkers for cardiometabolic risk.

Amino acids play a crucial role in various physiological processes, including protein synthesis, intracellular metabolism, and immune response [25]. Six out of seven of the included reports documented an association of amino acids with changes in BMI and insulin resistance [15,16,18,19,21]. Among the overarching class of amino acids, peptides, and analogs included were glycylproline, Citrulline, formiminoglutamic acid, 4-hydroxyproline, alanine, phenylalanine, tyrosine, glutamine, methionine, serine, and alanine. In 2016, Zhao et al. reviewed insulin resistance in childhood obesity using blood metabolomics studies [26]. The authors concluded that amino acid and lipid metabolism were the most impacted. Specifically, branched-chain amino acids (BCAAs), aromatic amino acids (AAAs), and acylcarnitines were identified as being closely associated with insulin resistance and potential future cardiometabolic risk. Analysis of the cord blood metabolome of 399 newborns from four European cohorts showed that lower levels of BCAAs, valine, and leucine predicted overweight in childhood [27]. Furthermore, Hellmuth et al. [16] showed that changes in acylcarnitines and amino acids following weight loss owing to the implementation of lifestyle interventions were associated with improved insulin sensitivity, supporting the notion that these metabolites may serve as indicators of metabolic improvements. These findings suggest that metabolic shifts resulting from weight loss could improve overall health by addressing imbalances in these biomarkers.

One of the most critical metabolic disturbances associated with obesity in children is insulin resistance and DM2. The relation between obesity and insulin resistance is complex, especially in children. Understanding the mechanisms underlying insulin resistance in childhood obesity is crucial. Metabolomic profiling is an emerging approach to investigate the molecular origins of insulin resistance in children. Various cross-sectional studies in animals and adults have shown associations between insulin resistance or DM2 and BCAAs, as well as aromatic amino acids (AAAs), sulfur-containing amino acids, other amino acids, and short-chain acylcarnitines (Carns) [28,29,30]. The study by Hellmuth et al. [16] demonstrated that tyrosine and AAAs are the only metabolites significantly associated with HOMA-IR at baseline and after one year of the implementation of a lifestyle intervention program inducing substantial weight loss > 0.5 BMI standard deviation (SD) scores in children with obesity. Similarly, in a meta-analysis of 8000 adults, there was a 36% higher risk of DM2 per study-specific SD difference for isoleucine, 35% for valine, 36% for tyrosine, and 26% for phenylalanine [31]. The authors explained that tyrosine is an aromatic amino acid metabolically linked to BCAAs and may act as a primary alteration or a more significant marker in the cascade of metabolic changes, even more consistently than the BCAAs, in some cases. Furthermore, Hellmuth et al. [16] used different ratios as biomarkers for insulin resistance, such as C5/C6-oxo, C4/C5-oxo, C6-oxo/xLeu, and C5-OH/C5:1 in different subgroups. All of these indicated that incomplete fatty acid oxidation was related to a higher score of HOMA-IR. It is likely that reduced complete fatty acid oxidation results in the stimulation of proinflammatory pathways, impaired insulin action in skeletal muscle, enhanced mitochondrial stress, and finally impaired glucose metabolism in humans and rodents. Furthermore, given that C3 and C5 acylcarnitines were the byproducts of BCAAs, reduced complete fatty acid oxidation seemed to be influenced by BCAA metabolism, indicating a close interaction of amino acid metabolism and lipid metabolism. In the study of Hosking et al. [21], insulin resistance was associated with a characteristic molecular phenotype, including lower levels of BCAAs and 2-ketobutyrate. This association was mainly attributed to the role of the mitochondrial enzyme Branched-Chain Keto acid Dehydrogenase (BCKD) in the generation of elevated BCAAs and branched-chain keto acids in insulin resistance and obesity [32]. The Krebs cycle through citrate and 3-hydroxybutyrate intermediates reduced ketogenesis, while elevated lactate and alanine concentrations were found to precede insulin resistance. The association between lactate and insulin resistance was robust even after adjusting for confounders, suggesting that lactate may serve as a reliable biomarker for metabolic health. Lactate, which is traditionally considered a byproduct of anaerobic metabolism, has emerged as an important molecule in metabolic disorders, and its elevated levels may reflect a shift toward anaerobic glycolysis, which is commonly seen in conditions of insulin resistance and metabolic stress. However, these findings are derived from a longitudinal study of a cohort of healthy children and may be different in children with obesity.

Obesity also represents a cardiovascular risk factor. One study evaluated childhood metabolic predictors of adult cardiovascular disease risk (MetS score) in a cohort of 396 females [20]. The authors suggested that exposure to atherogenic Apolipoprotein profile and low HDL concentrations in childhood, as well as a proinflammatory response that includes GlycAs, may lead to changes in the arteries that contribute to the development of atherosclerosis and coronary heart disease in adulthood. The ApoB/ApoA1 ratio indicates the balance between atherogenic ApoB and antiatherogenic ApoA1 cholesterol particles and is strongly and positively correlated with cardiovascular risk in adults [33]. In a pediatric population, the ratio of ApoB/ApoA1 ratio was also strongly correlated with increased waist circumference, BMI, fat percentage, diastolic blood pressure, and incidence of MetS [34]. Furthermore, the ApoB/ApoA1 ratio in young Finns predicted carotid intima-media thickness and brachial endothelial function in adulthood [35]. The association of low HDL phospholipids with coronary artery disease has been documented in adult subjects too [36]. Finally, GlycAs might also serve as biomarkers of subclinical vascular inflammation. In the study by Mansell et al. [19], decreases in BMI were associated with decreases in glycoprotein acetyls. In a cross-sectional study of 9842 adults, GlycAs correlated with adiposity, insulin resistance, and other markers of metabolic syndrome and all-cause mortality [37,38].

The present study has notable strengths. One of the key strengths of this systematic review is its comprehensive approach to literature search and inclusion criteria, which ensured a broad capture of relevant studies. An additional strength is the diversity of the included populations, spanning multiple countries and age groups. Moreover, the focus on longitudinal studies and randomized controlled trials with long follow-up periods allows for a more in-depth understanding of causal relations and the progression of metabolic changes associated with obesity.

Despite its strengths, the review has several limitations. The potential for publication bias is significant, given that studies with positive findings are more likely to be published. This bias may overestimate the association between certain metabolites and obesity outcomes. In addition, the exclusion of non-English studies may limit the comprehensiveness of the review, as relevant research published in other languages was not considered. The included studies varied widely in their design, sample size, population characteristics, and methodologies. This heterogeneity introduces variability in the results, making direct comparisons between studies challenging. There is a lack of standardization in the metabolomic techniques and analytical platforms used across the included studies. Differences in sample preparation, metabolite extraction, and data analysis can lead to inconsistencies in the identification and quantification of metabolites. This variability may impact the reproducibility of the findings. The wide age range of participants, from early childhood to adolescence, may introduce age-related metabolic variations that are not fully accounted for in the analyses. Metabolic processes can differ significantly between younger children and adolescents, affecting the generalizability of the results. The studies included in this review were conducted in various geographic regions, including Europe, the USA, and Australia. While this diversity is a strength, it also introduces potential geographic and socioeconomic variations that may influence metabolic profiles. Differences in diet, lifestyle, and access to healthcare can impact the generalizability of the findings.

The results from our systematic review suggest that changes in BMI in children and adolescents with obesity are associated with specific metabolic changes in the metabolome of individuals, especially in amino acids and lipid metabolism, which may serve as powerful diagnostic tools for disease monitoring and risk assessment. Understanding the pathways and mechanisms driving these associations is crucial for developing targeted interventions. Further research should focus on elucidating these mechanisms to provide a more comprehensive understanding of the metabolic alterations associated with obesity. In the future, longitudinal studies that track metabolic changes over time, intervention studies that evaluate the efficacy of therapeutic strategies, and large-scale population studies that explore metabolic diversity across different demographics will be important.

## 5. Conclusions

Metabolomics enhances our comprehension of disease progression and metabolic pathways in an obesogenic environment. This systematic review offers valuable insights into specific metabolite patterns characteristic of childhood obesity, including metabolically healthy and unhealthy phenotypes, and potential metabolomic profiles associated with their complications. However, available longitudinal studies in children and adolescents are few, and further studies are required to confirm the proposed metabolic signature.

Weight loss appears to improve several metabolic parameters, including lipid metabolism, amino acid profiles, and inflammatory markers, which may contribute to better insulin sensitivity and reduced risk for metabolic diseases. Notably, certain metabolites such as glycoprotein acetyls, ApoB/ApoA1 ratio, and lactate emerge as promising biomarkers for insulin resistance and metabolic syndrome, suggesting their potential utility in clinical settings. The metabolic fingerprints gleaned from these studies not only shed light on the current state of metabolic health in children with obesity but also have the potential to guide preventive and therapeutic strategies. This knowledge can be particularly important in the pediatric population, where early intervention might have long-lasting effects. Given the findings, the high risk of bias in some studies does not undermine the overall value of metabolomics in childhood obesity research but emphasizes the need for stringent methodological approaches in future studies to enhance the reliability of the conclusions drawn. As such, continued exploration of metabolomic profiles in childhood obesity is warranted, particularly in pediatrics, to develop targeted interventions and prevent the long-term consequences of this condition.

## Figures and Tables

**Table 1:** PICO/PECO framework for study selection on metabolomic biomarkers and childhood obesity risk.

Variable	Definition
Population	Children and adolescents aged 2–19 years
Exposure/Intervention	Metabolomics, metabolic signatures, and metabolic biomarkers
Comparator	No intervention, any intervention, or standard care The absence of the exposure or a different level of exposure
Outcome	Ov/Ob and MUO risk Association of metabolic signatures/biomarkers with obesity/adiposity/metabolic disorders/endocrine disorders

**Table 2:** Inclusion and exclusion criteria.

Parameter	Inclusion Criteria for All Domains	Exclusion Criteria for All Domains
Participants	Human subjects	Animals Human subjects with monogenic disorders (e.g., MC4R deficiency, leptin deficiency, etc.), syndromic forms of obesity (e.g., Prader–Willi, Alstrom syndrome, etc.), or subjects receiving medication known to affect weight (antidepressants, antiepileptics, antipsychotics, mood stabilizers, antimanic agents, and corticosteroids)
Age	2 to 19 years old	<2 years old and >19 years old
Article type	Peer-reviewed journal articles	Letters, editorials, study or review protocols, pre-prints
Study area	Europe, USA, Canada, Oceania	Asia, Africa, South America
Study design	Longitudinal prospective studies, randomized controlled trials with ≥12 months of follow-up, and meta-analyses of the above	Cross-sectional studies, controlled experiments, in vitro studies, in vivo animal studies, in silico studies, and scoping reviews
Time of publication	1 January 2013–3 July 2024 for original publications and 1 January 2018–3 July 2024 for meta-analyses	Original publications prior to 31 December 2012 and meta-analyses prior to 31 December 2017
Language	English	Non-English

**Table 3:** Characteristics of the included studies.

Study	Country	Study Design	Sample Size	Age, Mean ± SD	Follow-Up Period	Methodology	Key Metabolites Identified
Singh et al., 2023 [18]	USA	Longitudinal cohort study (Buckeye Teen Health Study)	81 (100% males)	16.08 ± 1.20 years	1 year	UPLC-QTOF-MS (urine)	Glycylproline, 3’-Sialyllactose, Formiminoglutamic acid, 4-hydroxyproline, Citrulline, Inosine
Mansell et al., 2022 [19]	Australia	Longitudinal cohort study (COBRA cohort)	98 (52% males)	10.3 ± 3.5 years	5 years	NMR (serum)	XL-VLDL-L, L-VLDL-L, S-VLDL-L, ApoB/ApoA1, VLDL-C, MUFAs, MUFAs%, alanine, phenylalanine, tyrosine, pyruvate, glycoprotein acetyls, HDL-C, LA%, Omega-6%, PUFAs, Acetoacetate, 3-hydroxybutyrate
Reinehr et al., 2014 [15]	Germany	Post hoc analysis of participants who underwent a lifestyle intervention (“Obeldicks”) within a non-randomized controlled trial	160 (61.3% males)	11 ± 2 years	1 year	HPLC-MS (serum)	Glutamine, methionine, LPCaC18:1, LPCaC18:2, LPCaC20:4, PCaeC36:2
Hellmuth et al., 2019 [17]	Europe (multi)	Post hoc longitudinal analysis of biomarker changes over 2.5 years in participants from the CHOP study, a double-blind, randomized intervention trial	396 (50% males)	5.5 ± 0.07 years	2.5 years	UPLC-QTOF-MS (serum)	Free carnitine, SM 32:2, SM 34:2, Carn 3:0
Ojanen et al., 2021 [20]	Finland	Longitudinal cohort study	396 (0% males)	11.2 ± 0.4 years	7.5 years	NMR (serum)	ApoB/ApoA ratio, GlycAs
Hellmuth et al., 2016 [16]	Germany	Post hoc analysis of participants who underwent a lifestyle intervention (“Obeldicks”) within a non-randomized controlled trial	80 (45% males)	11.5 ± 2.4 years	1 year	HPLC-MS (serum)	Acylcarnitines, amino acids
Hosking et al., 2019 [21]	UK, Switzerland	Longitudinal cohort study (EarlyBird cohort)	190 [Study 1: 40 (50% males); Study 2: 150 (70% males)]	4.8–5.1 years	Study 1: 9 years; Study 2: 11 years	1H NMR (serum)	Amino acids, lipids, lactate

**Table 4:** Outcomes of interest, statistical analysis, and results of included studies.

Author, Year (Reference)	Outcomes	Statistical Analysis	Results
Singh et al., 2023 [18]	Significant metabolic features associated with positive change in BMI at 1-year follow-up.	Estimate (95% CI) based on a stratified linear regression model (age, race, BMI z-score, and total energy intake).	Glycylproline: −0.018 (−0.029, 0.007) *p* = 0.002, 3’-Sialyllactose: 0.009 (0.002, 0.016) *p* = 0.006, formiminoglutamic acid: 0.016 (0.004, 0.028) *p* = 0.008, glycylproline: −0.014 (−0.025, 0.003) *p* = 0.01, 4-hydroxyproline: 0.016 (0.003, 0.03) *p* = 0.016, Citrulline: 0.01 (0.002, 0.018) *p* = 0.013, 4-Vinylsyringol: −0.01 (−0.02, 0.001) *p* = 0.022, Citrulline: 0.012 (0.001, 0.023) *p* = 0.025, Inosine: 0.005 (0.0004, 0.01) *p* = 0.03,
Mansell et al., 2022 [19]	Association of change in BMI from baseline to the end of follow-up (5.5 years) with the change in metabolomic profiles.	Coefficients (95% CI) [Benjamini–Hochberg adjusted *p*-value] of the change in log concentrations of metabolites in SD units decrease in BMI over time per unit (kg/m ^2^) from linear regression models adjusted for age at each time point and sex.	lipoprotein subclasses: XL-VLDL-L: −0.038 (−0.066 to −0.01), *p* = 0.04; L-VLDL-L: −0.038 (−0.066 to −0.01), *p* = 0.04; S-VLDL-L: −0.039 (−0.071 to −0.008), *p* = 0.05; Apolipoproteins: ApoB/ApoA1: −0.046 (−0.073 to −0.019), *p* = 0.01; cholesterols: VLDL-C: −0.035 (−0.062 to −0.008), *p* = 0.05; HDL-C: 0.045 (0.011 to 0.08), *p* = 0.04; HDL2-C: 0.049 (0.016 to 0.082), *p* = 0.02; fatty acids: unsaturation: 0.059 (0.022 to 0.097), *p* = 0.02; MUFAs: −0.041 (−0.068 to −0.014), *p* = 0.02; LA%: 0.065 (0.03 to 0.101), *p* = 0.01; Omega-6%: 0.069 (0.034 to 0.103), *p* = 0.003; PUFAs%: 0.065 (0.03 to 0.1), *p* = 0.01; MUFAs%: −0.061 (−0.094 to −0.028), *p* = 0.01; amino acids: alanine: −0.072 (−0.105 to −0.04), *p* = 0.002; phenylalanine: −0.069 (−0.102 to −0.037), *p* = 0.002; tyrosine: −0.068 (−0.099 to −0.037), *p* = 0.002; glycerides and phospholipids: total triglycerides: −0.043 (−0.069 to −0.016), *p* = 0.02; VLDL-TGs: -0.042 (−0.07 to −0.015), *p* = 0.02; TG/PG: −0.052 (−0.081 to −0.023), *p* = 0.01; glycolysis-related metabolites: pyruvate: −0.077 (−0.114 to −0.039), *p* = 0.002; ketone bodies: Acetoacetate: 0.065 (0.021 to 0.109), *p* = 0.02; 3-hydroxybutyrate: 0.066 (0.018 to 0.113), *p* = 0.04; inflammation: glycoprotein acetyls: −0.063 (−0.092 to −0.035), *p* = 0.002. Additional adjustment for pubertal status confirmed statistically significant associations for fatty acids: LA%, PUFAs%, and MUFAs%; amino acids: alanine, phenylalanine, and tyrosine; glycolysis-related metabolites: pyruvate; and inflammation: glycoprotein acetyls.
Reinehr et al., 2014 [15]	Change in metabolites between groups (children with obesity with substantial weight loss and children with obesity without weight loss; all underwent a lifestyle intervention).	The 14 metabolites [glutamine, methionine, proline, nine phospholipids (PCaeC34:1, C34:2, C34:3, C36:2, C36:3, C38:2, LPCaC18:1, C18:2, and C20:4), and two acylcarnitines (C12:1 and C16:1)] were compared between baseline and 1-year follow-up.	The 14 metabolites did not change significantly in children without weight loss. In children with substantial weight loss, glutamine [mean (SD) at baseline: 567 (120), follow-up: 588 (102), *p* = 0.013], methionine [mean (SD) at baseline: 27 (6), follow-up: 29 (6), *p* = 0.026], LPCaC18:1 [mean (SD) at baseline: 10 (2.8), follow-up: 10.9 (3), *p* = 0.003], LPCaC18:2 [mean (SD) at baseline: 12.3 (5.2), follow-up: 13.5 (5.2), *p* = 0.035], LPCaC20:4 [mean (SD) at baseline: 19.6 (8.2), follow-up: 21.7 (7.7), *p* = 0.011] and PCaeC36:2 [mean (SD) at baseline: 4.5 (1.7), follow-up: 4.8 (1.4), *p* = 0.026] increased significantly, while the other eight metabolites did not change significantly.
Hellmuth et al., 2019 [17]	Researchers used the metabolite concentrations at 5.5 years to predict the BMI z-score at 8 years of age in the CHOP study.	Linear regression models adjusted for child age and gender.	Plasma levels of free carnitine (*p* = 6.17 × 10^−6^), SM 32:2 (*p* = 2.16 × 10^−4^), SM 34:2 (*p* = 3.09 × 10^−4^) and Carn 3:0 (*p* = 4.09 × 10^−2^) were significantly positively associated with the BMI z-score at 8 years of age. However, after adjusting for the BMI z-score at 5.5 years, no metabolite reached the significance level. Regarding HOMA, glutamine at age 5.5 years was significantly negatively associated (*p* = 0.013/0.003) with HOMA indices at 8 years in both the unadjusted and adjusted linear models. NEFAs 26:1 (*p* = 0.012/0.015), 26:2 (*p* = 0.002/0.01), and 26:3 (*p* = 0.009/0.015) at age 5.5 years were significantly positively associated with HOMA indices at 8 years in both the unadjusted and adjusted linear models. Only serine levels remained significantly associated with HOMA in the adjusted model (*p* = 0.032).
Ojanen et al., 2021 [20]	To assess cardiometabolic risk, a standardized continuously distributed variable for clustered metabolic risk (MetS score) was constructed. The risk score was calculated by standardizing and then summing the following continuously distributed metabolic traits: mean arterial pressure ([(2 × diastolic blood pressure) + systolic blood pressure]/3); abdominal fat mass; fasting plasma glucose; serum HDL cholesterol x −1; and fasting serum triglyceride z-score. The z-scores for each variable and MetS scores were calculated separately for each time point. A higher score indicated a higher cardiometabolic risk.	Regression analysis with MetS score as the dependent variable and metabolic biomarkers identified by LASSO as independent variables, after Bonferroni correction for multiple tests.	Baseline ApoB/ApoA ratio and GlycAs positively predicted while L-HDL-PLs negatively predicted 7.5-year Mets (r = 0.471 and *p* < 0.0001; r = 0.400 and *p* = 0.0005; and r = −0.465 and *p* < 0.0001, respectively, *p*: adjusted for multiple comparisons by Bonferroni). And 2-year ApoB/ApoA ratio and GlycAs positively predicted and L-HDL-PLs negatively predicted 7.5-year Mets (r = 0.449 and *p* < 0.0001; r = 0.440 and *p* < 0.0001; and r = −0.445 and *p* < 0.0001, respectively, *p*: adjusted for multiple comparisons by Bonferroni) only. ApoB/ApoA ratio, GlycAs, and L-HDL-PLs remained significant predictors of MetS score (*p* < 0.0001 for all). These associations were also robust to multi-covariate adjustment, including insulin, leptin, adiponectin, sex steroids, IGF-1, physical activity, and energy yield nutrient intakes.
Hellmuth et al., 2016 [16]	Association of changes in metabolite concentrations with change in HOMA over the one-year intervention.	Change was defined as the relative change over the one-year intervention, with estimates reported alongside 95% confidence intervals (CIs). To assess the association between metabolites and markers of insulin resistance, a two-step robust regression approach was used. First, metabolite levels were adjusted for BMI using age- and sex-adjusted robust regression (M-estimator with Huber bi-square weighting). The residuals from this model were then regressed on the relative change in HOMA over the intervention period, using robust regression to minimize the influence of outliers.	All: Carn C0 1.10 [0.29; 1.90] *p* = 0.008, Carn C6:1-DC −0.33 [−0.59; −0.06] *p* = 0.015, Carn C6-oxo −0.24 [−0.43; −0.05] *p* = 0.014, Pro 0.81 [0.19; 1.40] *p* = 0.011; ratio of Carn C5/Carn C6-oxo 0.24 [0.07; 0.41] *p* = 0.007, ratio of Carn C6-oxo/xLeu −0.19 [−0.34; −0.03] *p* = 0.016, Tyr 0.79 [0.17; 1.40] *p* = 0.015; weight loss: AAA sum 1.04 [0.29; 1.80] *p* = 0.009, Carn C0 1.71 [0.88; 2.50] *p* < 0.001, Carn C3 0.49 [0.03; 0.96] *p* = 0.036; Carn C6:1-DC −0.29 [−0.47; −0.10] *p* = 0.003; Carn C6-oxo −0.21 [−0.35; −0.08] *p* = 0.003, Pro 0.72 [0.11; 1.30] *p* = 0.023, ratio of Carn C4/Carn C5-oxo 0.48 [0.18; 0.77] *p* = 0.0030, ratio of Carn C5/Carn C6-oxo 0.22 [0.08; 0.35] *p* = 0.002, ratio of Carn C6:1-DC/Carn C5:1 −0.22 [−0.41; −0.03] *p* = 0.024, ratio of Carn C6-oxo/xLeu −0.15 [−0.25; −0.04] *p* = 0.007, Trp 1.13 [0.14; 2.10] *p* = 0.027, Tyr 1.09 [0.51; 1.70] *p* = 0.001, Val 0.73 [0.07; 1.40] *p* = 0.033; no weight loss: ratio of Carn C5/Carn C6-oxo 0.29 [0.02; 0.57] *p* = 0.041.
Hosking et al., 2019 [21]	Association between individual metabolites and IR (HOMA-IR), taking into account age, BMI z-scores, and physical activity. Study 1 was designed as a pilot study to explore whether HOMA-IR was associated with specific metabotypes. Study 2 aimed at replicating the observations in a higher number of children, and extending the analysis to the age of 16 years.	Coef (SE), Bonferroni-adjusted *p*-value of mixed effects models for the association between the metabolite and log IR over time. Adjusted for age, gender, BMI z-score, APHV (age at peak height velocity), MVPA (number of minutes spent in moderate-vigorous physical activity), and individual metabolites.	Study 1: Leucine [−0.103 (0.027), *p* = 0.01]; Valine [−0.107 (0.026), *p* = 0.003]; 3-D-hydroxybutyrate [−0.106 (0.027), *p* = 0.01]; alanine [0.085 (0.024), *p* = 0.03]; 3-D-hydroxybutyrate [−0.084 (0.024), *p* = 0.02]; citrate [−0.132 (0.028), *p* = 0.0002]; Creatine [−0.095 (0.029), *p* = 0.06]; phospholipids [−0.131 (0.031), *p* = 0.002]; Study 2: lipids (mainly LDL, fatty acid CH3 moieties) [0.108 (0.023), *p* = 0.0006]; Leucine [−0.121 (0.019), *p* = <0.0001]; Valine [−0.114 (0.02), *p* = <0.0001]; 2-Ketobutyrate [−0.071 (0.019), *p* = 0.024]; 3-D-hydroxybutyrate [−0.092 (0.018), *p* = <0.0001]; lipids (mainly LDL, fatty acids [CH2]n moieties) [0.133 (0.023), *p* = 0.00093]; lactate [0.101 (0.019), *p* = <0.0001]; alanine [0.156 (0.019), *p* = <0.0001]; lipids (mainly VLDL, fatty acids [CH2] moieties) [−0.12 (0.022), *p* = <0.0001]; Arginine [−0.116 (0.021), *p* = <0.0001]; Lysine [−0.112 (0.019), *p* = <0.0001]; glutamate [−0.112 (0.021), *p* = <0.0001]; 3-D-hydroxybutyrate [−0.118 (0.019), *p* = <0.0001]; glutamine [−0.118 (0.022), *p* = <0.0001]; citrate [−0.188 (0.021), *p* = <0.0001]; Asparagine [−0.115 (0.021), *p* = <0.0001]; Trimethylamine [−0.123 (0.022), *p* = <0.0001]; Dimethylglycine [−0.118 (0.021), *p* = <0.0001]; Lysine [−0.139 (0.02), *p* = <0.0001]; Creatine [−0.142 (0.023), *p* = <0.0001]; Citrulline [−0.137 (0.022), *p* = <0.0001]; Creatine [−0.121 (0.025), *p* = 0.00013]; serine [−0.134 (0.022), *p* = <0.0001]; Histidine [−0.136 (0.021), *p* = <0.0001]; Histidine [−0.124 (0.022), *p* = <0.0001]. When correcting for main covariates (gender, age, BMI z-scores, physical activity, and APHV), only the association between lactate and log IR remained significant.

Abbreviations: APHV—at peak height velocity; ApoB/ApoA—Apolipoprotein B to Apolipoprotein A-1; BMI—Body Mass Index; Carn—acylcarnitine; CI—confidence interval, GlycAs—glycoprotein acetyls; HOMA—Homeostatic Model Assessment for Insulin Resistance; IGF-1—insulin-like growth factor 1; IR—insulin resistance; LASSO—Least Absolute Shrinkage and Selection Operator; L-HDL-PLs—large high-density lipoprotein phospholipids; LPC—lyso-phosphatidylcholine; MUFAs—monounsaturated fatty acids; NEFAs—non-esterified fatty acids; *p*—*p*-value; PCae—alkyl–acyl phosphatidylcholine; PUFAs—long-chain polyunsaturated fatty acids; SE—standard error; SM—sphingomyelins; VLDL—very-low-density lipoprotein.

**Table 5:** Risk of bias of the included studies.

Study [Author, Year (Reference)]	Risk of Bias for Longitudinal Studies							
	D1	D2	D3	D4	D5	D6	D7	Overall
Singh et al., 2023 [13]	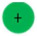	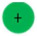	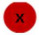	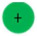	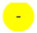	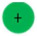	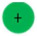	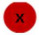
Mansell et al., 2021 [14]	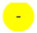	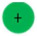	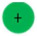	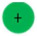	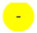	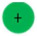	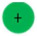	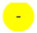
Reinehr et al., 2015 [15]	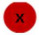	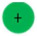	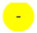	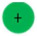	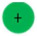	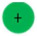	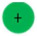	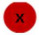
Hellmuth et al., 2019 [16]	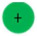	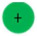	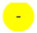	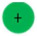	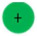	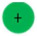	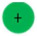	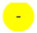
Ojanen et al., 2021 [18]	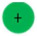	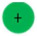	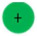	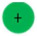	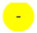	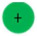	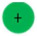	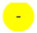
Hellmuth et al., 2016 [21]	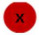	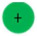	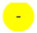	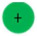	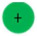	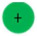	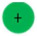	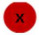
Hosking et al., 2019 [19]	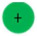	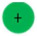	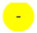	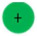	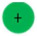	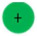	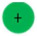	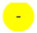

Notes: Domain of risk of bias, i = 1–7 as follows. D1, due to confounding. D2, arising from the measurement of the exposure. D3, in the selection of participants in this study (or in the analysis). D4, due to post-exposure interventions. D5, due to missing data. D6, arising from the measurement of the outcome. Color/symbol coding of risk of bias. 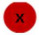; high risk of bias. 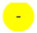; some concerns. 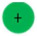; low risk of bias.

## Data Availability

Not applicable.

## Supplementary Materials

The following supporting information can be downloaded at https://www.mdpi.com/article/10.3390/nu17111833/s1. File S1. Search strings used for MEDLINE (PubMed) and Scopus.
