# Supplementary material for: Exploring Metabolic Signatures: Unraveling the Association with Obesity in Children and Adolescents
Source: Nutrients. 2025 May 28;17(11):1833. doi: 10.3390/nu17111833 (PMC12157888; doi:10.3390/nu17111833)
Supplement: Supplementary file 1 [file nutrients-17-01833-s001.zip › nutrients-3618280-supplementary.pdf]

SUPPLEMENTARY MATERIAL

S1: Search strings used for MEDLINE (PubMed) and Scopus

Keywords for PubMed: *"((""metabolomics""[Title/Abstract] OR ""metabolic signatures""[Title/Abstract] OR ""metabolic biomarkers""[Title/Abstract]) AND (""weight""[Title/Abstract] OR ""body weight""[Title/Abstract] OR ""body mass index""[Title/Abstract] OR ""BMI""[Title/Abstract] OR ""BMI percentile""[Title/Abstract] OR ""BMI z-score""[Title/Abstract] OR ""overweight""[Title/Abstract] OR ""obesity""[Title/Abstract] OR ""child* overweight""[Title/Abstract] OR ""child* obesity""[Title/Abstract] OR ""adipos*""[Title/Abstract] OR ""body fat""[Title/Abstract] OR ""fatness""[Title/Abstract] OR ""skinfold thickness""[Title/Abstract] OR ""waist circumference""[Title/Abstract] OR ""fat mass""[Title/Abstract] OR ""fat free mass""[Title/Abstract] OR ""muscle mass""[Title/Abstract] OR ""neck circumference""[Title/Abstract] OR ""waist-to-height""[Title/Abstract] OR ""body composition""[Title/Abstract] OR ""weight gain""[Title/Abstract] OR ""weight loss""[Title/Abstract] OR ""weight status""[Title/Abstract] OR ""weight change""[Title/Abstract] OR ""metabolically unhealthy obesity""[Title/Abstract] OR ""*hypertension""[Title/Abstract] OR ""blood pressure""[Title/Abstract] OR ""dyslipidaemia""[Title/Abstract] OR ""dyslipidemia""[Title/Abstract] OR ""hyperlipidaemia""[Title/Abstract] OR ""hyperlipidemia""[Title/Abstract] OR ""hypercholesterolaemia""[Title/Abstract] OR ""hypercholesterolemia""[Title/Abstract] OR ""blood lipid*""[Title/Abstract] OR ""cholesterol""[Title/Abstract] OR ""triglyceride*""[Title/Abstract] OR ""blood glucose""[Title/Abstract] OR ""glycated haemoglobin""[Title/Abstract] OR ""glycated hemoglobin""[Title/Abstract] OR ""glycaemic control""[Title/Abstract] OR ""glycemic control""[Title/Abstract] OR ""HOMA""[Title/Abstract] OR ""HOMA-IR""[Title/Abstract] OR ""insulin resistance""[Title/Abstract] OR ""prediabetes""[Title/Abstract] OR ""metabolic syndrome""[Title/Abstract] OR ""inflammation""[Title/Abstract] OR ""liver enzymes""[Title/Abstract] OR ""hepatic steatosis""[Title/Abstract] OR ""non-alcoholic fatty liver disease""[Title/Abstract] OR ""NAFLD""[Title/Abstract] OR ""polycystic ovary syndrome""[Title/Abstract] OR ""PCOS""[Title/Abstract] OR ""obstructive sleep apnoea""[Title/Abstract] OR ""obstructive sleep apnea""[Title/Abstract] OR ""OSA""[Title/Abstract] OR ""uric ac-id""[Title/Abstract] OR ""hyperuricaemia""[Title/Abstract] OR ""hyperuricemia""[Title/Abstract])) AND (""child*""[Title/Abstract] OR ""adolescen*""[Title/Abstract] OR ""teen""[Title/Abstract] OR ""teenage*""[Title/Abstract] OR ""preteen*""[Title/Abstract] OR ""youth""[Title/Abstract] OR ""paediatric""[Title/Abstract] OR ""pediatric""[Title/Abstract] OR ""juvenile*""[Title/Abstract] OR ""youngster*""[Title/Abstract] OR ""girl""[Title/Abstract] OR ""girls""[Title/Abstract] OR ""boy""[Title/Abstract] OR ""boys""[Title/Abstract] OR ""kid""[Title/Abstract] OR ""kids""[Title/Abstract] OR ""young person*""[Title/Abstract] OR ""young people""[Title/Abstract] OR ""school-child*""[Title/Abstract] OR ""school age""[Title/Abstract] OR ""schoolage*""[Title/Abstract] OR ""student*""[Title/Abstract] OR ""pupil*""[Title/Abstract])",,,from 2013/1/1 - 3000/12/12,"((""metabolomics""[Title/Abstract] OR ""metabolic signatures""[Title/Abstract] OR ""metabolic biomarkers""[Title/Abstract]) AND (""weight""[Title/Abstract] OR ""body weight""[Title/Abstract] OR ""body mass index""[Title/Abstract] OR ""BMI""[Title/Abstract] OR ""BMI percentile""[Title/Abstract] OR ""BMI z-score""[Title/Abstract] OR ""overweight""[Title/Abstract] OR ""obesity""[Title/Abstract] OR ""child overweight""[Title/Abstract] OR ""child obesity""[Title/Abstract] OR ""adipos*""[Title/Abstract] OR ""body fat""[Title/Abstract] OR ""fatness""[Title/Abstract] OR ""skinfold thickness""[Title/Abstract] OR ""waist circumference""[Title/Abstract] OR ""fat mass""[Title/Abstract] OR ""fat free mass""[Title/Abstract] OR ""muscle mass""[Title/Abstract] OR ""neck circumference""[Title/Abstract] OR ""waist-to-height""[Title/Abstract] OR ""body composition""[Title/Abstract] OR ""weight gain""[Title/Abstract] OR ""weight loss""[Title/Abstract] OR ""weight status""[Title/Abstract] OR ""weight change""[Title/Abstract] OR ""metabolically unhealthy obesity""[Title/Abstract] OR ""hypertension""[Title/Abstract] OR ""blood pressure""[Title/Abstract] OR ""dyslipidaemia""[Title/Abstract] OR ""dyslipidemia""[Title/Abstract] OR ""hyperlipidaemia""[Title/Abstract] OR ""hyperlipidemia""[Title/Abstract] OR ""hypercholesterolaemia""[Title/Abstract] OR ""hypercholesterolemia""[Title/Abstract] OR ""blood lipid*""[Title/Abstract] OR ""cholesterol""[Title/Abstract] OR ""triglyceride*""[Title/Abstract] OR ""blood glucose""[Title/Abstract] OR ""glycated haemoglobin""[Title/Abstract] OR ""glycated hemoglobin""[Title/Abstract] OR ""glycaemic control""[Title/Abstract] OR ""glycemic control""[Title/Abstract] OR ""HOMA""[Title/Abstract] OR ""HOMA-IR""[Title/Abstract] OR ""insulin resistance""[Title/Abstract] OR ""prediabetes""[Title/Abstract] OR ""metabolic syndrome""[Title/Abstract] OR ""inflammation""[Title/Abstract] OR ""liver enzymes""[Title/Abstract] OR ""hepatic steatosis""[Title/Abstract] OR*

""non-alcoholic fatty liver disease""[Title/Abstract] OR ""NAFLD""[Title/Abstract] OR ""polycystic ovary syndrome""[Title/Abstract] OR ""PCOS""[Title/Abstract] OR ""obstructive sleep apnoea""[Title/Abstract] OR ""obstructive sleep apnea""[Title/Abstract] OR ""OSA""[Title/Abstract] OR ""uric acid""[Title/Abstract] OR ""hyperuricaemia""[Title/Abstract] OR ""hyperuricemia""[Title/Abstract]) AND (""child""[Title/Abstract] OR ""adolescen*""[Title/Abstract] OR ""teen""[Title/Abstract] OR ""teenage*""[Title/Abstract] OR ""preteen*""[Title/Abstract] OR ""youth""[Title/Abstract] OR ""paediatric""[Title/Abstract] OR ""pediatric""[Title/Abstract] OR ""juvenile*""[Title/Abstract] OR ""youngster*""[Title/Abstract] OR ""girl""[Title/Abstract] OR ""girls""[Title/Abstract] OR ""boy""[Title/Abstract] OR ""boys""[Title/Abstract] OR ""kid""[Title/Abstract] OR ""kids""[Title/Abstract] OR ""young person*""[Title/Abstract] OR ""young people""[Title/Abstract] OR ""schoolchild*""[Title/Abstract] OR ""school age""[Title/Abstract] OR ""schoolage*""[Title/Abstract] OR ""student*""[Title/Abstract] OR ""pupil*""[Title/Abstract])) AND (2013/1/1:3000/12/12[pdat])"; Language: English, Filter year: 2013and onwards.*

Keywords for Scopus: *TITLE-ABS-KEY (("child*" OR "adolescen*" OR "teen" OR "teenage*" OR "preteen*" OR "youth" OR "paediatric" OR "pediatric" OR "juvenile*" OR "youngster*" OR "girl" OR "girls" OR "boy" OR "boys" OR "kid" OR "kids" OR "young person*" OR "young people" OR "schoolchild*" OR "school age" OR "schoolage*" OR "student*" OR "pupil*") AND (("metabolomics" OR "metabolic signatures" OR "metabolic biomarkers"))) AND (LIMIT-TO (DOCTYPE , "ar") OR LIMIT-TO (DOCTYPE , "re")) AND (LIMIT-TO (LANGUAGE , "English")) AND TITLE-ABS-KEY ("weight" OR "body weight" OR "body mass index" OR "BMI" OR "BMI percentile" OR "BMI z-score" OR "overweight" OR "obesity" OR "child* overweight" OR "child* obesity" OR "adipos*" OR "body fat" OR "fatness" OR "skinfold thickness" OR "waist circumference" OR "fat mass" OR "fat free mass" OR "muscle mass" OR "neck circumference" OR "waist-to-height" OR "body composition" OR "weight gain" OR "weight loss" OR "weight status" OR "weight change" OR "metabolically unhealthy obesity" OR "*hypertension" OR "blood pressure" OR "dyslipidaemia" OR "dyslipidemia" OR "hyperlipidaemia" OR "hyperlipidemia" OR "hypercholesterolaemia" OR "hypercholesterolemia" OR "blood lipid*" OR "cholesterol" OR "triglyceride*" OR "blood glucose" OR "glycated haemoglobin" OR "glycated hemoglobin" OR "glycaemic control" OR "glycemic control" OR "HOMA" OR "HOMA-IR" OR "insulin resistance" OR "prediabetes" OR "metabolic syndrome" OR "inflammation" OR "liver enzymes" OR "hepatic steatosis" OR "non-alcoholic fatty liver disease" OR "NAFLD" OR "polycystic ovary syndrome" OR "PCOS" OR "obstructive sleep apnoea" OR "obstructive sleep apnea" OR "OSA" OR "uric acid" OR "hyperuricaemia" OR "hyperuricemia") AND (LIMIT-TO (DOCTYPE , "ar") OR LIMIT-TO (DOCTYPE , "re")) AND (LIMIT-TO (LANGUAGE , "English"))*
